# Social and Structural HIV Prevention in Alcohol-Serving Establishments: Review of International Interventions Across Populations

**Published:** 2010

**Authors:** Seth C. Kalichman

**Keywords:** Alcohol and other drug use, sexually transmitted infection, human immunodeficiency virus, acquired immune deficiency syndrome, drinking venue, bars, pubs, taverns, high-risk sexual behavior, unsafe sex, high-risk populations, gay men, female sex workers, prevention, intervention, preventive interventions

## Abstract

Alcohol use is associated with risks for sexually transmitted infections (STIs), including HIV/AIDS. People meet new sex partners at bars and other places where alcohol is served, and drinking venues facilitate STI transmission through sexual relationships within closely knit sexual networks. This paper reviews HIV prevention interventions conducted in bars, taverns, and informal drinking venues. Interventions designed to reduce HIV risk by altering the social interactions within drinking environments have demonstrated mixed results. Specifically, venue-based social influence models have reduced community-level risk in U.S. gay bars, but these effects have not generalized to gay bars elsewhere or to other populations. Few interventions have sought to alter the structural and physical environments of drinking places for HIV prevention. Uncontrolled program evaluations have reported promising approaches to bar-based structural interventions with gay men and female sex workers. Finally, a small number of studies have examined multilevel approaches that simultaneously intervene at both social and structural levels with encouraging results. Multilevel interventions that take environmental factors into account are needed to guide future HIV prevention efforts delivered within alcohol-serving establishments.

Alcohol use is among the most reliable predictors of sexual risk behavior across populations at high risk for human immunodeficiency virus (HIV)/acquired immune deficiency syndrome (AIDS). Associations between alcohol use and sexual risks are observed regardless of whether alcohol use is measured in terms of global patterns of drinking, or within situations where drinking occurs, or at the level of sexual events (Weinhardt and Carey 2001). Alcohol elevates sexual risks through multiple channels, including the psychological effects of intoxication on decisionmaking, inhibition of protective actions, interactions between drinking and risk-taking personality characteristics, and perceived expectations regarding the effects of alcohol use on risk-taking and sexual experiences (Cook and Clark 2005). The association between drinking and sexual risk behaviors has led to interventions that seek to reduce both alcohol use and sexually risky behaviors (Palepu et al. 2005). For example, an HIV risk reduction intervention for inner-city African-American men reduced alcohol use in sexual contexts by 85 percent from baseline (Kalichman et al. 1999). In a similar study of one-on-one risk reduction counseling in South Africa, researchers reported a 59 percent reduction in alcohol use in sexual contexts 3 months following the intervention (Kalichman et al. 2007). However, the intervention effects on sex-related alcohol use decreased within 6 months to a 43 percent reduction from baseline. This short-lived effect is likely the result of individuals returning to unchanged high-risk environments that support risk-related alcohol use (Kincaid 2004), suggesting the need for interventions to alter risk factors within alcohol-serving environments.

This article examines the alcohol-serving venues that intersect with HIV transmission risks and offer potential environments for HIV prevention. Venues of greatest interest are those that sell alcohol for onsite consumption and serve as a point of social interaction. Multiple types of drinking establishments fit these criteria across cultures, including neighborhood bars, dance clubs, karaoke bars, taverns, wine shops, and informal lounges. In addition to describing the intersection of drinking places with HIV/AIDS risks, this article reviews the literature on HIV prevention interventions based in alcohol venues. Venue-based interventions are defined as strategies designed to alter the social and/or physical environment to promote protective action and reduce risk. Therefore, this article does not include interventions that target drinkers or those that are delivered outside of drinking places. The outcomes from completed venue-based intervention trials point to gaps in the existing literature and suggest future directions for HIV prevention efforts based in alcohol-serving venues.

## Alcohol-Serving Establishments and HIV Transmission Risks

The contextual factors within which alcohol and sexual behaviors intersect are critical to our understanding of how alcohol influences HIV risks. The environments of drinking establishments are multifaceted and serve multiple functions, including being a place for recreation, fostering socially sanctioned drinking, facilitating social relationships, and providing opportunities to meet sex partners. The subsequent sections review selected studies that describe sexual risks for HIV incurred within drinking places. These studies are summarized in Table 1.

### Studies of Gay Men

Early studies of HIV risks in alcohol-serving establishments were focused on gay bars, primarily in the United States and Europe. Across studies, approximately one in three men who have sex with men sampled from gay bars and clubs reported engaging in unprotected anal intercourse, the highest risk behavior for HIV transmission. Nardone and colleagues (2001), for example, found that one-third of men who go to gay bars in London and Edinburgh engaged in unprotected anal sex, and only half of the men had been tested for HIV. A study in California showed that one in three men who visit gay clubs and bars reported five or more sex partners in the past year (Xia et al. 2006). Men who socialized at gay bars also demonstrated higher risk for HIV infection than gay men who did not go to bars, although men who drank at bars were at lower risk than men who attended sex-focused “circuit” parties. Another study showed that men who meet sex partners at gay bars may not differ significantly from men who meet partners at other open gay venues, such as bathhouses, private sex parties, gyms, and the Internet (Grov et al. 2007). Although behaviors across gay community venues may not differ, the prevalence of disease does vary. In China, for example, HIV (0.8 percent) and syphilis (2.4 percent) prevalence in gay bars is substantially lower than in gay saunas (3.6 percent and 20.7 percent, respectively) (Hong et al. 2009). From an HIV prevention perspective, gay bars may therefore be primarily social venues where sex partners meet and drinking may merely be a contextual rather than causal factor.

### Other High-Risk Populations

Commercial sex workers represent a risk population that also is frequently accessed in drinking places. Female sex workers in Malawi, for example, who drink and contract a sexually transmitted infection (STI) are less likely to use condoms than their counterparts who also contract STIs but do not drink (Zachariah et al. 2003). Women who socialize in drinking places, whether involved in sex work or not, demonstrate a high risk for HIV infection (Kapiga et al. 2002; Singh et al. 2009). Meeting sex partners in drinking places increases the likelihood of sex after drinking and reduces condom use (Mataure et al. 2002; Sivaram et al. 2008).

### Analyzing the Overlap of Drinking Venues and Sex-Partner Meeting Places

Concurrency of sex partners within and outside of drinking places is a significant facet of HIV/AIDS in southern Africa (Carter et al. 2007; Mah and Halperin 2008) and is facilitated by the sexual networks within drinking establishments. Sex partners often meet at informal alcohol-serving establishments that are common throughout southern Africa, such as private homes where alcoholic beverages are sold and served (Morojele et al. 2006; Weir et al. 2003). For example, one study (Kalichman et al. 2008) that surveyed men and women recruited from informal local township bars (e.g., shebeens) in South Africa found that more than one in four drinkers reported having met sex partners at their local shebeen. People who meet sex partners in drinking venues report greater numbers of recent sex partners and higher rates of unprotected intercourse compared with people who did not meet partners in drinking places. Only half of those surveyed had been tested for HIV in a community in South Africa where one in five people are HIV infected (Shisana et al. 2005). These patterns of risk create urgency for HIV prevention in drinking venues.

Using a well-developed system for rapid community assessments, Weir and colleagues (2003) mapped the linkages among places where people meet new sex partners and places where people drink alcohol. Studies that have used Weir’s PLACE methodology show a consistent overlap among drinking behaviors and sexual risks in alcohol-serving venues (Figueroa et al. 2007; Khan et al. 2008). For example, in Zimbabwe, 47 percent of women ages 15–19 years surveyed at night clubs and other drinking places had two or more sex partners in a 1-year period compared with less than 5 percent of young women surveyed outside of drinking places (Singh et al. 2009). In South Africa, more than 85 percent of the places where people meet sex partners are alcohol-serving venues (Weir et al. 2003).

The overlap between drinking places and venues where people meet sex partners was observed in both urban and rural areas. Across three cities in South Africa, between 78 percent and 87 percent of new sex partners met at shebeens. As many as half of the people who drink at shebeens report having had two or more sex partners in the previous 2 weeks, and more than half of men and one in five women who have met partners in shebeens have had sex on the premises (Kalichman et al. 2008). Not surprisingly, informal alcohol-serving establishments rarely display HIV prevention messages or have condoms available for their customers (Figueroa et al. 2007; Weir et al. 2003).

### Homes Where Alcohol Is Sold

Alcohol also is often sold from lounges in people’s homes that double as small alcohol-serving businesses. People who live in alcohol-serving home businesses are at increased risk for HIV/AIDS. In Ugandan villages, for example, only 4 percent of people live in homes that serve alcohol, but the HIV prevalence in these homes is 15 percent, nearly double that of the surrounding community (Mbulaiteye et al. 2000).

### Beer Halls

HIV risks also are observed in larger drinking venues, such as taverns and beer halls. Basset and colleagues (1996) reported that the HIV prevalence in Zimbabwean beer halls (i.e., large social venues that primarily serve men) is as much as twice that of the general Zimbabwe population. Sexual mixing patterns within beer halls facilitate HIV transmission, especially when other STIs are prevalent. For example, 60 percent of men and 40 percent of women who have multiple sex partners in Zimbabwe drink at beer halls (Lewis et al. 2005). Fritz and colleagues (2002) found similar behaviors in these venues and showed that HIV prevalence increases with greater frequency of drinking at beer halls.

### Drinking Venue Employees

Employees as well as patrons of alcohol-serving establishments are at higher risk for HIV infection. For example, men who work in Tanzanian bars and hotels who drink at least once a week are significantly more likely to have herpes simplex virus infection, a known marker for HIV transmission risk, than other men surveyed at the same places who do not drink (Kapiga et al. 2002, 2003). Women working in food and recreational businesses near southern-African gold mines who drink are significantly more likely to have HIV infection than other women who drink in the communities that surround the mines but do not work in drinking venues (Clift et al. 2003).

### Social and Sexual Networks

Higher rates of unprotected sex and STIs in contexts with high HIV prevalence create highly favorable circumstances for HIV transmission. HIV can rapidly spread within drinking venues that have tightly knit social and sexual networks which foster overlapping sexual mixing patterns. Alcohol- serving establishments amplify HIV transmission risks by providing a place where sex encounters occur with rapid turnover of partners (Fritz et al. 2002). Alcohol establishments are often themselves sex venues, where back rooms, back corners, and adjacent buildings or shacks offer locations for sex (Morojele et al. 2006). Beyond the social interactions that naturally occur in drinking places, the use of alcohol itself and its association with sexual risk behaviors makes these environments compelling venues for HIV risk reduction. The social and physical structures of alcohol drinking places therefore create opportunities for community-based HIV prevention interventions.

## Alcohol Venue–Based HIV Prevention

A review of the literature on HIV interventions delivered within alcohol-serving establishments yielded studies including randomized trials, quasi-experimental studies (i.e., interventions in which the intervention groups and control groups were not randomized), and public health evaluations. The interventions evaluated in these studies (summarized in Table 2) targeted diverse populations in the United States, the United Kingdom, Asia, and Africa and concentrated on altering the social and/or physical environments of drinking places to reduce HIV transmission risks. For the most part, the target populations were consistent with the HIV risk populations of the countries. Specifically, gay bars were targeted in the United States and United Kingdom, whereas female sex workers were the focus in Asia, and in Africa the target has been drinking places that serve local communities within generalized HIV epidemics. The interventions are characterized by their (1) targeting social interactions, (2) changing structural and environmental features, and (3) targeting both social and structural levels.

### Social Influence Interventions

The majority of alcohol venue–based interventions target social environments to influence behavior. Such interventions aim to change the perception that a particular behavior is socially acceptable (i.e., alter the perceived social norms) and influence the interactions of people in the environment to shape and sustain protective social norms.

Of nine trials identified, five focused on social influences for HIV prevention within drinking venues. Four interventions explicitly tested the popular opinion leader (POL) model (Kelly et al. 1997), and one evaluated a peer-led intervention that approximated the central features of the POL model. The POL model identifies, trains, and motivates key community-defined opinion leaders to act as agents of change by shifting social norms and thereby behaviors toward safer practices. The POL intervention is grounded in diffusion of innovation theory (Rogers 2003), which specifies that trends and innovations are initiated by a relatively small segment of a population that acts as natural opinion leaders within environmentally or geographically demarcated populations. Innovations that are adopted, modeled, and endorsed by opinion leaders diffuse across social gradients throughout a population. Opinion leaders are trained in interpersonal communication skills to enhance their sense of self-efficacy for acting as agents of change. Although generalized to other settings, the POL model was designed for use in drinking places in small cities, where bars may offer the only outlet for gay men to socialize.

The POL intervention requires a small cadre of trusted, well-liked people who frequent the bar to be trained to initiate conversations and endorse safer sex practices. The active ingredient in the intervention is therefore informal casual conversations with peers, rather than formal education. Using conversations in natural settings as the basis for delivering the intervention is the most compelling feature of the POL model. In these conversations, POLs can correct misinformation; discuss the importance of HIV in the community; talk with network members about strategies to reduce risk, such as keeping condoms nearby; avoiding sex when intoxicated; and resisting unsafe sex. The intervention uses environmental prompts to serve as conversation starters, such as posters or t-shirts with messages and logos. The intervention core elements are (1) identifying and enlisting the support of popular and well-liked opinion leaders to take on risk-reduction advocacy roles, (2) training cadres of opinion leaders to disseminate risk-reduction endorsement messages within their own social networks, and (3) supporting and reinforcing successive waves of opinion leaders to help shape social norms to encourage safer sex (Kelly 2004).

### POL Studies Set in Small U.S

#### Cities

Kelly and colleagues (1992) reported significant reductions in high-risk sexual practices in smaller cities in the southern United States resulting from the POL intervention. The study used a quasi-experimental design with multiple baselines and staggered intervention implementation. The first city to receive the POL intervention had two baselines and three 3-month (9 months)-interval postintervention implementation followups. Two additional cities implemented the intervention after four baselines, with one city having two (6 months) and the other having one (3 months) postintervention followup. The pattern of results was consistent across the three cities: the POL intervention demonstrated reductions in men reporting multiple sex partners, unprotected insertive and receptive anal sex, and increased condom use. The magnitude of change was significant, with up to 29 percent reductions from baseline levels. These behavior changes were bolstered by theoretically predicted changes in perceived social norms supportive of safer sex.

Following the quasi-experimental study, Kelly and colleagues (1997) conducted an experimental test of the community-level POL intervention with eight matched pairs of smaller U.S. cities randomized to either receive the POL program or a comparison intervention that placed general HIV prevention education materials and condoms in the bars. The intervention implementation in half of the pairs of cities was systematically confounded with changes in season, possibly playing havoc with the intervention implementation and assessments. The outcomes were therefore based on the remaining four pairs of cities. The findings mostly replicate the behavior changes observed in the earlier quasi-experimental study, demonstrating 30 percent reductions in unprotected anal intercourse and 50 percent increases in condom use.

### POL Study Set in New York City

A study that implemented the POL model in New York City successfully replicated Kelly and colleagues’ (1992) quasi-experimental trial (Miller et al. 1998, 2003) among an extremely high-risk group of male sex workers (i.e., hustlers). The adapted intervention demonstrated significant reductions in paid sex encounters and unprotected anal sex in paid sexual relations. In addition, moderator analyses showed that White and Latino men reduced their frequency of unpaid unprotected anal intercourse in response to the intervention, with no such reduction observed among Black men. This pattern of results was similar to that observed by Kelly and colleagues (1992), although with a somewhat smaller effect size.

### POL With Community Interventions

As part of a larger multicountry trial (National Institute of Mental Health [NIMH] 2008), researchers adapted the POL intervention to include additional elements of community mobilization derived from non–bar-based community-level HIV prevention interventions (Kegeles et al. 1996). The adapted version was called the community–POL intervention, or C–POL. Researchers conducted the C–POL intervention in five countries, and in one country the implementation occurred in alcohol-serving establishments. Specifically, the intervention was delivered at wine shops in slums of Chennai, India. Wine shops serve mostly beer and distilled alcohol as well as light food and have bars attached to them where patrons can sit and socialize. Such shops primarily serve men, although women frequently meet men outside or near the wine shops. A cluster of wine shops served as the defined intervention venue. The intervention group demonstrated substantial reductions in unprotected sex behaviors and reductions in STIs over the 2-year observation period (NIMH Collaborative HIV/STD Prevention Trial 2008). However, similar reductions in risk and disease were observed in wine shops that served as controls, where only nontailored prevention messages were delivered. Specifically, wine shops exposed to the C–POL intervention had an STI incidence of 7 percent compared with 8 percent in the nonintervention wine shops. Therefore, results of the C–POL intervention on wine shop patrons in India did not demonstrate significant effects on HIV transmission risk behaviors or on biologically assessed STIs.

#### Peer-Led Intervention

Fritz and colleagues (2009) implemented a social influence intervention that targeted men who socialize and drink in Zimbabwean beer halls. The intervention used a peer-led education model that emphasized men talking with each other about HIV/AIDS prevention. Men volunteered to attend a 3-day training to learn about HIV and how they can help their friends avoid HIV infection. The training included elements of information/education, motivational enhancement, and behavioral skills training for HIV risk reduction. Men who became “peer educators” agreed to volunteer and deliver prevention messages for 15 months posttraining. Like the POL/C–POL model, this intervention was aimed at diffusing prevention strategies and messages throughout the beer hall to change social norms and behavior. Twelve beer halls were randomly assigned to receive either the peer influence intervention or a standard message and access to condoms. The intervention failed to demonstrate any significant effects on behavioral risk indicators, including unprotected sex acts within and across specific partner types, condom use, and numbers of partners. For example, men in the beer hall intervention sites reduced their unprotected sex by 11.8 episodes compared with a reduction of 15 episodes among men in the control venues. The reductions were both significant but not significantly different from each other. Similarly, men in the intervention beer halls reported an average of five episodes of unprotected intercourse with nonwife partners, as did the men in the control beer halls.

### Structural Interventions

Interventions that aim to influence behavior through changes in law, policy, and the physical environment generally are referred to as structural interventions (Blankenship et al. 2000). In HIV prevention, examples of structural interventions include mandatory HIV testing for sex workers, closing of public sex environments, and legal needle and syringe exchange programs. Structural interventions also can include improved services that are meant to avert HIV transmission, such as enhanced STI detection and treatment and improved HIV prevention services (Bauermeister et al. 2008). Only two studies have reported on the use of structural approaches to HIV prevention in alcohol-serving establishments. Both studies were conducted as public health program evaluations that did not include comparison conditions. Rou and colleagues (2007) implemented strategies to enhance HIV/STI education and diagnostic and treatment services for female sex workers in bars, massage parlors, dance halls, and beauty parlors in two provinces in China. The intervention included active outreach to women in the venues as well as condom promotion campaigns. Over 1 year of observation, the program showed significant improvements in HIV prevention knowledge, reductions in unprotected sex, and increased condom use among women across venues. Prevalence of incident STIs dropped dramatically during the study period.

In a similar approach taken with men who have sex with men in New York City, Blank and colleagues (2005) reported the effects of the “hot shot” public health program. Aimed at men in gay bars, the intervention offered STI screening and diagnostic and treatment services in alcohol venues. The program reached over 1,600 men in six clubs and three community-based organizations and delivered health services to between 12 percent and 44 percent of event attendees. The program was deemed successful in terms of its implementation and detection of new cases of STIs and HIV. However, changes in behavior or in disease outcomes were not reported.

#### Combining Social Influence and Structural Interventions

A potentially powerful approach to HIV prevention in alcohol-serving establishments is the combination of both social influence and structural interventions. These multilevel models attempt to simultaneously shift social norms to reinforce protective behaviors as well as enhance services and change policies toward HIV prevention. Two studies reported the effects of multilevel social and structural interventions delivered in drinking places. Flowers and colleagues (2002) tested the Gay Men’s Task Force intervention in Glasgow, Scotland. This intervention had three major components, one social influence and two structural, each of which had been shown effective in the past and combined for the first time. The intervention included peer-led sexual health promotion conducted in five gay bars by 42 peer educators over a 9-month period. Peer educators, modeled after opinion leaders in the POL intervention, received 2 days of training focused on communication skills for promoting safer sex messages. A second component of the intervention established enhanced STI and sexual health services for gay men as well as a free sexual health hotline service. Results from the two-city quasi-experimental trial demonstrated a significant effect for engaging men in STI services, as evidenced by increased hepatitis B vaccination and HIV testing. Among men who had conversations with peer educators, 49 percent had contemplated and 26 percent reported making changes in their sexual behaviors. However, there was no evidence of community-wide sexual behavior changes resulting from the intervention (Hart et al. 2004).

The second multilevel intervention targeted women sex workers in bars, discos, and night clubs in the Philippines (Morisky et al. 2006). The social influence intervention component was grounded in peer counseling. Female sex workers were enlisted and trained in HIV risk education and prevention actions, focusing on condom use and sexual negotiation skills. The structural component of the intervention enlisted the managers of the drinking places to attend HIV education sessions. The managers were trained to implement a continuum of HIV prevention policies and practices. The training focused on promoting employee health, with specific emphasis on the diagnosis and treatment of STIs. The quasi-experimental study used cities as the unit of randomization, with four cities randomized to four conditions. One site received the peer-counseling intervention, one site received the structural manager intervention, a third site received both the peer-influence and manager components (multilevel condition), and the fourth condition received neither component. Results showed positive effects for both the individual peer counseling and manager components. However, the combination of peer counseling and manager training demonstrated the greatest impact on condom attitudes, venue policies favoring condom use, and reductions in subsequent STIs over the 2-year observation period.

### Summary of Intervention Findings

Alcohol venue–based HIV prevention interventions demonstrate a mixed pattern of results. Social influence interventions, particularly the POL model, appear effective in U.S. gay bars, including bars in small and large cities. What these venues have in common are close-knit social networks within which community members know and often trust each other. Gay communities have few public places where people can meet and socialize. Although the social landscape is changing in gay communities (Simon Rosser et al. 2008), gay bars historically have been the focal point. Still, social influence models closely tied to the POL model did not work in U.K. gay bars, Indian wine shops, and Zimbabwean beer halls. Understanding the characteristics of social networks and environments that account for the effects of social influence interventions will determine the ultimate utility of these approaches.

The structural interventions reviewed here demonstrated promising outcomes. Enhancing STI and sexual health services through venue-based programming increased hepatitis vaccination and HIV testing in the U.K. and reduced STIs in women involved in sex work in China and men attending gay bars in New York City. Most promising are the additive effects of social influence and manager training to improve sex-worker health in the Philippines. The pattern of results from these few trials suggest that interventions that simultaneously target individual, social, and structural risk factors may hold the greatest promise for alcohol venue–based HIV prevention.

## Advancing Alcohol Venue–Based HIV Prevention

Models of HIV risk reduction focused on the individual may be insufficient for reducing alcohol-related HIV risks because they do not address social, structural/environmental, and contextual influences on behavior. Individual-level models rarely achieve sustained behavior change due to unaltered risk environments, the potency of peer pressure, social reinforcements for maintaining risk behaviors, and the interaction between alcohol use and alcohol environments in promoting sexual risks. Interventions that address social influences on behavior, such as social norms and interpersonal contingencies of reinforcement (such as in the POL model), will help support initial behavior changes for potentially long durations. In addition, changes to the physical environment, including policies that impact behavior, also can maintain risk-reducing practices. Interventions that integrate individual, interpersonal, and environmental levels of change will likely prove most effective in reducing alcohol-related HIV risks.

Individual-level intervention components aim to influence motivation and build problem-solving skills, condom use, and sexual negotiation skills. Individual skills-based HIV prevention interventions have proven effective at reducing HIV risk behaviors among drinkers, albeit for short periods of time (Kalichman et al. 2007). Individual skills-building interventions also can include communication skills training geared toward influencing social interactions and social networks within drinking environments. Finally, environmental and policy interventions can support the individual and social influence changes by creating more protective environments. Although there are few structural interventions for sexual risk behavior, policies that regulate drinking age, maintain safety standards of drinking environments, offer health services to sex workers, and provide universal access to STI treatment will likely have a significant impact on HIV risks.

Multilevel alcohol venue–based interventions are feasible for implementation in alcohol-serving establishments. Weir and colleagues (2008) describe environmental components that fit well within the parameters of multilevel interventions. Social cues and prompts can be placed in drinking environments by using posters to communicate prevention messages. Condoms also can be made accessible in venues using dispensers and condom machines with signage. Weir and colleagues (2008) suggest using outreach workers with brief surveys to help engage patrons and raise awareness. Finally, events that support HIV prevention, such as drama and music events, can be implemented in drinking places and include onsite HIV testing and STI screening. Structural changes in the drinking environment will both foster and sustain behavior change that occurs with intensive interventions delivered at individual and social levels. As suggested by Morisky and colleagues (2006), structural changes can be implemented gradually and in synch with the current environment and policies.

HIV prevention behaviors can be adopted, accepted, and become normative within social networks (Kelly et al. 2006; Latkin et al. 2004), and these processes can occur within drinking environments. A subset of influential community members can encourage behavior change through informal social interactions in existing relationships within drinking places. Concepts derived from network-based interventions can be applied though multilevel approaches and provide a mechanism for altering social environments to shape, support, and reinforce behavior change. Interventions can be designed to use existing social networks in drinking places. Peer-driven interventions, for example, that have been successful in U.S. gay bars and among injection drug using populations can be enhanced by having community health and outreach workers deliver prevention messages and resources, such as condoms. Drinking environments can provide venues for chain recruitment of successive waves of peers. At the structural level, drinking environments can post prevention messages that encourage conversation. Venue owners can be enlisted to help shape the house rules, enforce drinking laws, provide condoms, invite health department screenings and onsite testing, and promote the health of their staff as well as clients.

Historically, HIV prevention science has examined intervention components one level at a time. Results from single-level interventions have been discouraging, whereas interventions implemented at multiple levels have been more encouraging. Thus, it would appear that the most promising approaches to effective alcohol venue–based interventions are those that are multilevel and sustained over long periods of time. Multilevel HIV prevention interventions in drinking venues therefore warrant further investigation.

## Figures and Tables

**Table 1:** Selected International Studies of HIV Risks in Alcohol-Serving Venues

**Authors**		**Samples**	**Summary of Findings**
Singh 2009		511 young women, 15–24 years old, Zimbabwe	Young women who socialize at drinking places demonstrated the greatest risk for STI/HIV, with 35 percent reporting two or more sex partners in the previous year (compared with 5 percent of women in other venues) and 30 percent reporting exchanging sex for money in the past year (compared with 10 percent of women in nonalcohol venues).
Hong et al. 2009		1,146 men in gay saunas, bars, and clinics, China	HIV and syphilis prevalence were higher in gay saunas than gay bars. Demographic characteristics also differed substantially across venues with younger, gay-identified men attending bars and older married men attending saunas.
Sivaram et al. 2008		1,196 male wine shop/bar patrons, India	Nearly one in four people had four or more sex partners, 89 percent used alcohol in conjunction with sex, and alcohol use was associated with unprotected sex.
Kalichman et al. 2008		91 men and 248 women who drink in informal bars (shebeens), South Africa	Participants who meet sex partners in the drinking establishment (28 percent) had higher risk for HIV infection compared with patrons who did not meet sex partners in shebeens. Men and women who meet sex partners in shebeens did not differ in their risk behaviors.
Grov et al. 2007		886 men who have sex with men recruited at venues where they meet sex partners, New York and Los Angeles	Exact venue where sex partners meet, including bars, bath houses, private sex parties, gyms, public areas, and the Internet did not relate to patterns of HIV risk behavior. Personal characteristics and identity were related to risk practices across venues.
Xia et al. 2006		398 men who have sex with men and meet sex partners in various venues, California	Three of four men visited a gay bar at least once in the previous year. Men who go to bars demonstrate high rates of sexual risk behavior compared with men who do not go to any gay venues and less risk than men who attend circuit parties
Zachariah et al. 2003		1,817 female sex workers, Malawi	Sex workers with symptomatic STIs who drank were four times more likely to not use condoms than were STI symptomatic women who did not drink (37 percent vs. 12 percent).
Weir et al. 2003	3,085 men, 1,564 women in alcohol venues in townships and business districts, South Africa		People in townships and business districts meet sex partners in drinking establishments and sexual risk is closely associated with venues where drinking occurs.
Kapiga et al. 2003	309 female and 206 male bar and hotel workers, Tanzania		Alcohol use by men once a week was associated with increased risk of STIs compared with nondrinkers, particularly risks for contracting herpes simplex virus.
Kapiga et al. 2002	312 women working in bars and hotels, Tanzania		Women who worked in bars demonstrated 26 percent HIV prevalence, with risk for HIV 2.5 times greater for women who drank 2 or more days per week.
Fritz et al. 2002	324 men attending beer halls, Zimbabwe		HIV prevalence increased with greater frequency of drinking. Eleven percent of men reporting no alcohol use were HIV positive as were 41 percent of men that drank on 15 or more of the last 30 days. HIV prevalence and incidence were related to meeting sex partners in beer halls and having sex while intoxicated.
Mataure et al. 2002	227 men and 78 women, 15 to 21 years old, at nightclubs and bottle stores, Zimbabwe		Sixty percent of young women at drinking establishments usually meet sex partners there. Forty-two percent had sex after drinking in the past 90 days.
Nardone et al. 2002	2,397 men who have sex with men from gay bars, London and Edinburgh		One-third of men practiced recent unprotected anal intercourse with more than half of men having been tested for HIV. Differences were observed between cities, with higher risk apparent in London.

NOTE: HIV, human immunodeficiency virus; STI, sexually transmitted infection.

**Table 2:** HIV Risk Reduction Interventions Based in Alcohol Venues in International Settings

**Authors**	**Target Population and Venues**		**Intervention and Study Design**	**Summary of Findings**
**Social Influence Interventions**				
NIMH Collaborative HIV/STD Prevention Trial 2008		Wine shop customers in impoverished slums, India	C-POL intervention; 24 wine shops within impoverished slum communities; community-level RCT conducted in India and four other countries; observation over 2 years.	Comparable reductions in HIV/STI transmission risk behaviors occurred in the intervention and control communities. Both conditions also demonstrated comparable reductions in new STI.
Miller 1998		Gay bars with significant male prostitution in New York City	Adaptation of POL intervention; multiple baseline quasi-experimental design with staggered intervention implementation; observations occurred in each bar five times at 2-month intervals.	The intervention demonstrated significant reductions in paid USI across venues. Frequencies of unpaid USI were not affected by the intervention. Bars with more stable patrons and Latino and White men benefited from the intervention, whereas Black men did not.
Kelly et al. 1997		Gay bars in eight small U.S. cities	POL intervention, community-level RCT; observation over 1-year postintervention.	Intervention cities demonstrated less USI than control cities by a factor of nearly four- fold. Condom use increased from 45 percent protected acts to 67 percent, with no increase occurring in the control cities.
Kelly et al. 1992		Gay bars in three southeastern U.S. small cities	POL intervention; multiple baseline quasi-experimental design with staggered intervention implementation; observations between 3 and 9 months postintervention.	The POL model intervention demonstrated 15 percent to 24 percent reductions in USI across all three cities. Similar outcomes occurred for numbers of sex partners and increases in condom use during anal sex.
**Structural Interventions**				
Rou et al. 2008		Female sex workers in bars, massage parlors, dance halls, and beauty parlors, China	Enhanced STI clinic services with STI/HIV education and sex worker condom promotion outreach; five sites in two provinces participated in the public health evaluation with no control condition; 1-year observation period.	Reductions in multiple risk indicators were observed across venues during the observation period. Condom use with last client increased from 55 percent to 67 percent, prevalence of gonorrhea declined from 26 percent to 4 percent, and prevalence of Chlamydia decreased from 41 percent to 26 percent.
Blank et al. 2005		Gay bars and clubs identified through syphilis case interviews, New York City	”Hot Shot” public health program offering screening, diagnostic, and referral services to men in venues during evening hours; uncontrolled public health evaluation; 1-year observation period.	Implementation of portable health services in venues resulted in four new syphilis and seven new HIV detections. Packaging STI and HIV services with general health programming was deemed feasible and potentially effective.
**Multilevel Social Influence and Structural Interventions**				
Morisky et al. 2006		Female sex workers in bars, nightclubs, discos, beer gardens, and karaoke bars, Philippines	Four conditions: peer counseling and social influence for HIV risk reduction, manager training to reinforce employee health and structural health improvement, peer counseling with manager training, no-intervention control condition; quasi-experimental design with venues nested in four cities randomized to one of four conditions; 2-year observation period.	Increases in condom use at last sex and reductions in self-reported STI occurred in the combined peer/manager intervention condition compared with the control site. These effects were bolstered by more positive condom attitudes and increased condom promotion within the venues. Sites receiving either the peer or manager interventions demonstrated variable patterns of risk reduction relative to the control site.
Flowers et al. 2002		Gay bars in Glasgow, Scotland	Peer-led sexual health promotion conducted in venues, gay-specific sexual health services in hospitals, and free phone-hotline service; two- city quasi-experimental trial; 3-year observation period.	Peer education and associated services failed to demonstrate reductions in USI and increases in knowledge of sex partner HIV status or negotiated safety. Increases in HIV testing and hepatitis B vaccination were observed for men with direct contact with the intervention, but not for the Glasgow community in general.

NOTE: C-POL, community popular opinion leader; HIV, human immunodeficiency virus; POL, popular opinion leader; RCT, randomized community trial; STI, sexually transmitted infection; USI, unprotected sexual intercourse.
